# Enzymatic and microenvironmental regulation in adenosine metabolism-mediated immunosuppression

**DOI:** 10.3389/fimmu.2025.1739983

**Published:** 2026-01-07

**Authors:** Chuang Li, Lifeng Chen, Zhihao Li, Ling Liang, Benyong Lou

**Affiliations:** 1 College of Materials and Chemical Engineering, Minjiang University, Fuzhou, China; 2 School of Laboratory Medicine, Hubei University of Chinese Medicine, Wuhan, China

**Keywords:** adenosine metabolism, coordinated intervention, enzymatic regulation, immunosuppression, tumor microenvironment

## Abstract

Adenosine (ADO), as an endogenous purine nucleoside, can regulate almost all aspects of tissue function. However, its abnormal accumulation in the tumor microenvironment (TME) induces immune tolerance and promotes tumor immune evasion by activating adenosine receptors (ADOR). Regulating ADO metabolism in the TME holds promise for ameliorating ADO-mediated immunosuppression and restoring antitumor immune responses. Extensive research has highlighted the pivotal role of ADO in tumor immune suppression and preclinical development of inhibitors targeting ADOR. However, systematic integration in ADO metabolism of microenvironmental influences, enzyme and protein regulation, and targeted intervention strategies through multiple pathways remain insufficient. This review systematically summarizes the key aspects of targeting ADO-mediated immunosuppression, including the feature of TME, enzymes involved in ADO metabolism (e.g., CD39/CD73/ADK/ADA), and ADOR interventions. Additionally, the necessity of comprehensively regulating ADO metabolism and the immune microenvironment through multi-level coordinated interventions is also explored, as well as the latest combined regulatory strategies. Moreover, the major challenges in current research on ADO metabolic regulation are also critically analyzed and the future research directions are proposed to address the dual challenges of ADO metabolic diversity and TME complexity, aiming to develop more precise and effective immunotherapeutic strategies.

## 1 Introduction

Adenosine (ADO), as an endogenous purine nucleoside, is involved in the regulation of multiple physiological and pathological processes, such as myocardial energy metabolism, vasodilation, and inflammation/trauma repair (1–4). Under physiological conditions, the concentration of extracellular ADO is low (0.05–0.2 μmol/L) (5), which exerts protective effects by suppressing excessive inflammatory responses and immune-mediated tissue damage (6). However, in pathological microenvironments such as solid tumors, factors like hypoxia, chronic inflammation, and nutrient deprivation, an anomalous aggregation of ADO strongly influenced by massive adenosine triphosphate (ATP) release, overexpression of extracellular nucleotide hydrolases, and decreased adenosine kinase (ADK) activity (7, 8). Under these circumstances, ADO concentrations can notably increase by 100 times. Accumulated ADO subsequently activates adenosine receptors (ADOR) to modulate the activity of different immune cells, including macrophages, T lymphocytes, natural killer (NK) cells, dendritic cells (DCs), and myeloid-derived suppressor cells (MDSCs) (9). Through this process, an immunotolerant tumor microenvironment (TME) was ultimately established, laying a favorable background for tumor immune evasion and progression (10, 11). With the widespread recognition of the critical role of the immune system in tumor occurrence and progression, the regulatory effects of ADO metabolism on anti-tumor immune responses have emerged as a research focus (12).

**Figure 1:**
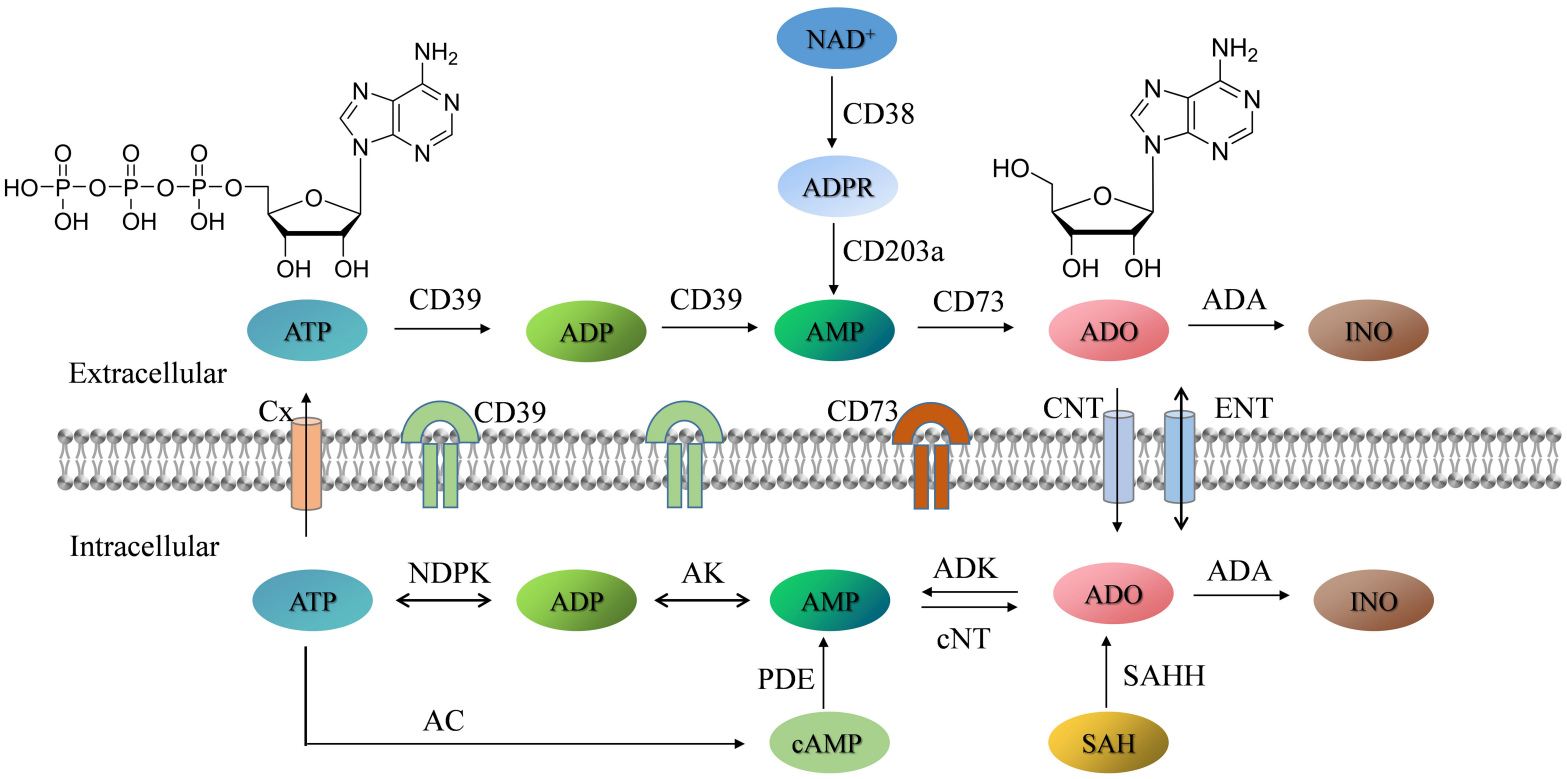
Schematic diagram of extracellular and intracellular ADO metabolism pathways. AC: Adenylate cyclase; ADP: Adenosine diphosphate; ADPR: Adenosine diphosphate ribose; AK: Adenylate kinase; AMP: Adenosine monophosphate; ATP: Adenosine triphosphate; cAMP: Adenosine 3’,5’-cyclic monophosphate; Cx: Connexin hemichannels; CNT/ENT: Nucleoside transporters; cNT: Cytosolic nucleotidase; INO: Inosine; NAD^+^: Nicotinamide adenine dinucleotide; NDPK: Nucleotide diphosphokinase; PDE: Phosphodiesterase; SAH: S-Adenosylhomocysteine; SAHH: S-Adenosylhomocysteine hydrolase.

ADO metabolism has high diversity and complexity, including all process of generation, transformation and degradation (10, 13, 14) (**Figure 1**). As mentioned before, the pathological microenvironments can trigger cell necrosis, apoptosis, and other secretory mechanisms, leading to the release of large amounts of ATP into the extracellular space. The extracellular ADO mainly originates from the hydrolysis of ATP by ectonucleoside triphosphate diphosphohydrolase (CD39) and ecto-5’-nucleotidase (CD73) located on the cell membrane (15, 16). Released ATP is first catalyzed by CD39 to produce adenosine monophosphate (AMP), which is subsequently hydrolyzed by CD73 into ADO. This classical metabolic progress from ATP through AMP to ADO forms the main pathway for extracellular ADO generation and plays a central role in regulating extracellular ADO level (17, 18). The other extracellular ADO comes from the release of intracellular ADO through passive diffusion or active transport. Additionally, nicotinamide adenine dinucleotide (NAD^+^) undergoes adenosine diphosphate (ADP) ribosylation reaction under the catalysis of CD38 (an NAD oxidase), producing ADP ribose (ADPR) or related intermediates. ADPR can then be further converted into AMP by CD203a or ectonucleotide pyrophosphatase/phosphodiesterase 1, and ultimately generate ADO via CD73-mediated catalysis (19, 20). By comparison, intracellular ADO metabolism is regulated by the coordinated action of multiple enzymes, including the process of phosphorylation, degradation, and endogenous generation. Under the catalysis of ADK, intracellular ADO can undergo phosphorylation reaction to generate AMP, reintegrated into the energy metabolism cycle (21). Under the catalysis of adenosine deaminase (ADA), ADO can be irreversibly degraded into inosine (INO) (22). For the intracellular ADO generation, it is endogenously generated through the dephosphorylation of AMP by cytoplasmic nucleotidase (cNT) (13). Moreover, S-adenosylhomocysteine (SAH) can also be hydrolyzed by SAH hydrolase (SAHH) to produce ADO and homocysteine, providing an alternative pathway for intracellular ADO generation and closely linked to cellular methylation reactions and epigenetic regulation (23).

In recent years, multiple authoritative reviews have systematically elaborated on the key advancements of ADO signaling and ADO-mediated immunosuppression in immunotherapy, demonstrating significant clinical translation potential (24–27). For instance, Luca Antonioli’s team elucidated the role of ADO and its receptors in regulating the complex interaction among immunity, inflammation, endothelial cells and cancer cells in the process of tumor disease (1). Detlev Boison’s group not only clarified the comprehensive mechanism of ADO metabolism but also emphasized the urgent need of the entire purine metabolome profiling for screening immunotherapy targets (13). Ling Ding’s team focused on the inhibitory effect and molecular mechanism of ADO on tumor adaptive immunity and summarized the clinical treatment progress of targeting the ADO pathway (28). These studies collectively promote the translation of ADO metabolism from fundamental mechanisms to clinical applications. However, systematic integration in ADO metabolism of microenvironmental influences, enzyme and protein regulation, and targeted intervention strategies through multiple pathways is still insufficient. This review systematically summarizes the key aspects of targeting ADO-mediated immunosuppression, including the feature of TME, enzymes involved in ADO metabolism (e.g., CD39/CD73/ADK/ADA), and ADOR interventions. The necessity of comprehensive regulation of ADO metabolism and the immune microenvironment through multi-level coordinated interventions is also explored. Furthermore, the major challenges in current research on ADO metabolic regulation have been thoroughly analyzed and the future research directions have been proposed. Given the dual challenges posed by the diversity of ADO metabolism and the complexity of TME, these efforts aim to address the difficulty of single target regulation in ADO-mediated immunosuppression, thereby providing the theoretical foundation for developing precise and effective immunotherapeutic strategies.

## 2 Signaling mechanisms of ADO-mediated immunosuppression

After being released into the extracellular space, ADO exerts immunomodulatory effects by binding to four G protein-coupled receptors (A1R, A2AR, A2BR, A3R) (29–31). These receptors are widely distributed in various tissues of the human body, but there are significant differences in affinity for ADO (32). A1R display high affinity and usually require 1–10 nM ADO concentration (33), which is the highest affinity among the four receptors. In contrast, A2AR, A2BR and A3R have lower affinity. Among them, A2BR has the lowest affinity for ADO (approximately 1000 nM) (34). A1R and A3R primarily transmit signals by inhibiting the formation of cAMP; whereas A2AR and A2BR participate in subsequent signal transduction through activation of AC and upregulation of cAMP levels that control the activity of various cells (35–38).

Under both physiological and pathological conditions, ADO primarily mediates immune regulation through the low-affinity receptors A2AR and A2BR (39). For example, ADO has specific immunomodulatory effects on the maturation, migration, and effector functions of NK cells. It markedly reduced their cytotoxic activity via A2AR signaling (40, 41), leading to tumor immune escape in several solid tumors by cAMP-dependent signaling that mediates protein kinase A (PKA) engagement (42). ADO also inhibits monocyte differentiation into macrophages through dual mechanisms of A2AR and A2BR, and promotes the polarization of macrophages from the pro-inflammatory M1 phenotype to the anti-inflammatory M2 phenotype (43). For DCs, ADO activates A2AR/A2BR on the surface of DCs and increases intracellular cAMP levels, which selectively target the protein kinase A/exchange protein activated by cAMP (PKA/EPAC) signaling pathway, leading to the inhibition of pro-inflammatory factor secretion (IL-12, TNF-α, IL-6, IL-8), and the promotion of anti-inflammatory/immunosuppressive factor release (IL-10, TGF-β, IDO, arginase 2, COX2), ultimately significantly reducing DCs antigen presentation ability (44, 45). At the T cell level, ADO impairs CD4^+^ T cell function and induces their conversion toward an immunosuppressive phenotype via A2AR and A2BR. ADO also inhibits CD8^+^ T cell proliferation, differentiation, and the production of effector cytokine through activating A2AR (46, 47). Among them, CD8^+^ T central memory cells (TCM) in the TME are particularly sensitive to ADO due to their high expression of A2AR. Elevated ADO in the TME continuously regulates TCM through A2AR, ultimately leading to functional failure of CD8^+^ T cells (48).

Furthermore, ADO can amplify the function of immunosuppressive cells by activating the forkhead box P3/lymphocyte activating 3 (FOXP3/LAG3) pathways via A2AR to promote regulatory T cell (Treg) differentiation (49, 50), and upregulating cytotoxic T-lymphocyte-associated protein 4 (CTLA-4) and programmed cell death protein 1 (PD-1) expression to strengthen the suppression of effector T cells. The accumulation of ADO has been confirmed to be the main mechanism by which Tregs exert immunosuppression (51). For myeloid cells, ADO enhances immunosuppressive activity of MDSCs through A2AR/A2BR via both the STAT3 and CREB signaling pathways, and contributes to tumor tissue remodeling and immunosuppressive microenvironment construction (52, 53).

Within the intricate immune regulation inherent the TME, ADOR expressed on specific cell surfaces is the pivotal molecular switch determining whether ADO transmits pro-tumor or anti-tumor signals. Collectively, the immunomodulatory effects of ADO are highly dependent on the differential expression of specific ADOR subtypes on various immune cells, thereby maybe mediating diametrically opposed biological functions (54). Among these, the A2A and A2B receptors are the primary subtypes responsible for mediating the immunosuppressive effects of ADO, particularly dominating in the immunosuppressive TME characterized by high ADO concentrations (55–57). Activation of this signaling pathway can inhibits the functionality of effector cells while activates immunosuppressive cells, ultimately blocking the immune response. Interestingly, the expression preferences of different immune cell subsets for specific receptor subtypes will provide precise entry points for development of A2AR and A2BR antagonists that target the intervention of ADO-mediated immunosuppression.

## 3 Strategies for targeting ADO-mediated immunosuppression

### 3.1 Targeting the hypoxic features of the tumor microenvironment

**Figure 2:**
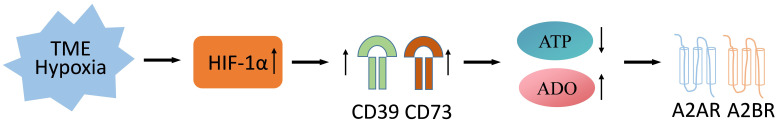
The hypoxic TME upregulates the expression of CD39 and CD73 via HIF-1α, accelerates the conversion of extracellular ATP into ADO, promotes the expression of A2AR and A2BR, and enhances the inhibitory effect of ADO on immune cells.

The immunosuppressive effect of TME is often driven by various abnormal features, among which hypoxia and acidity are the two most representative characteristics. Many solid tumors exhibit obvious insufficient local oxygen supply and persistent or chronic hypoxia due to rapid proliferation and vascular abnormalities, ultimately drives ADO-mediated immunosuppression (10, 58, 59). Under hypoxic condition, the expression of the hypoxia-inducible factor 1α (HIF-1α) significantly upregulates, leading to transcriptional activation of CD39 and CD73 expression levels (by 6 ± 0.5-fold compared to normoxic levels) (60, 61). This regulatory effect accelerates the extracellular conversion of ATP to ADO (**Figure 2**). Simultaneously, by upregulating the expression of A2AR and A2BR on the surface of immune cells, hypoxia also enhances the ADO-induced immunosuppressive signal (62–64). Furthermore, hypoxia extends the extracellular half-life of ADO by inhibiting ADK activity and the expression of nucleoside transporters (65, 66). These mechanisms collectively enable the hypoxic TME not only to convert pro-inflammatory ATP into ADO, but also to amplify ADO’s immunosuppressive function by prolonging action time and enhancing receptor sensitivity. Consequently, targeting the hypoxic TME becomes an effective entry point to block the abnormal accumulation of ADO in the TME.

Oxygenation strategies reshapes the “physiological oxygenation” microenvironment by improving tumor oxygenation levels in the TME, thereby reconstructing a “normoxic” microenvironment. It is reported that the supplemental oxygenation reprograms the hypoxic proteome and metabolome of tumors, prevents the inhibition of T cells and NK cells and decreases Treg cell populations, ultimately enhancing antitumor immune rejection (67). Respiratory hyperoxia therapy disrupts the immunosuppressive microenvironment by reducing the degree of tumor hypoxia and concentration of extracellular ADO, reversing the hypoxia-adenosinergic immunosuppression in the TME. The main manifestations were evidenced by elevated pro-inflammatory cytokines, reduced inhibitory molecules like TGF-β, and suppressed Treg cell activity (68). Additionally, iron-based compounds catalyze the generation of oxygen from excess H_2_O_2_ through Fenton-like reactions (69, 70), alleviating hypoxia and inhibiting ADO production. By regulating ADO levels, iron-based compounds can activate antitumor immune responses with increasing T cells and DCs while reducing Treg infiltration (71). In short, these interventions targeting hypoxia block the generation of ADO from the source, relieve microenvironmental immunosuppression, and enhance the antitumor efficacy of effector cells.

### 3.2 Targeting the acidic features of the tumor microenvironment

It is worth noting that the microenvironmental abnormalities of TME are not only manifested as hypoxia, but also accompanied by characteristic acidification. Under physiological conditions, the extracellular acid-base state of most normal tissues remains relatively stable, with pH value typically between 7.3 and 7.4. TME exhibits weak acidity (pH 6.7-7.1) due to lactate accumulation, inadequate perfusion, and uncontrolled proliferation caused by the Warburg effect of tumor cells (72, 73). This feature also has obvious regulatory influence on ADO metabolism and immune function. Under acidic conditions, the enzyme activity and expression levels of CD39 and CD73 different from those under neutral conditions (74–76), affecting the cascading hydrolysis of extracellular ATP/ADP to AMP and subsequently to ADO. In addition, HIF-1α can be activated by the dual stimuli of hypoxia and acidic microenvironment (77), increasing the production of ADO and amplifying the immunosuppressive state. Some studies have shown that the acidic microenvironment can inhibits ADO degradation by affecting the stability or substrate binding efficiency of ADA (78, 79). Meanwhile, by modulating the expression or function of purine metabolism-related transporters, the acidic characteristics indirectly influence the intracellular and extracellular distribution and dynamic equilibrium of ADO (80–82). All these above mechanisms confirm that targeting the acidic characteristics of the TME provides enormous potential for regulating ADO metabolism.

Existing studies have demonstrated that pH-balancing agents (83, 84) such as sodium bicarbonate, imidazole, and lysine can effectively inhibit tumor metastasis by neutralizing tumor-derived acidic substances. The acidic TME has also motivated the development of a variety of pH-responsive nanomaterials (85, 86). For example, Liu’s group designed polydopamine nanocarrier loaded with A2AR inhibitor coated by an acidic TME sensitive PEG shell to target the negative feedback of ADO-A2AR metabolic pathway. It weakens the metabolic inhibition of ADO and enhances the immune response of immunogenic cell death by promoting DC activation, increasing CD8^+^ T lymphocyte infiltration, and reducing MDSCs number (87). Similarly, the acid-responsive phosphatidylcholine-coated nanoparticles with poly-l-histidine core were constructed to achieve the release of the encapsulated CPI-444 in acidic TME and reverse Glioblastoma immunosuppressive microenvironment by targeting the ADO-A2AR pathway (88). Additionally, the engineered biohybrid Bc@AZTF was designed to interference of ATP-ADO Axis, which can actively enrich in tumor sites and respond to the acidic TME, consuming intracellular ATP content while inhibiting the ATP-ADO axis to reduce ADO accumulation, thereby alleviating ADO-mediated immunosuppression (89). These pH-balancing agents and pH-responsive nanomaterials employ differentiated mechanisms including direct neutralization, targeted release, or microenvironment regulation, to target the acidic TME. They not only achieve dynamic balancing or directional regulation of acidic substances but also provide a crucial intervention window for subsequent immunosuppressive therapy targeting ADO metabolism. By improving the acidic microenvironment of the TME, the generation of ADO can be blocked and the degradation efficiency of ADO can be improved, thereby reversing the immunosuppressive state.

### 3.3 Targeting enzymatic metabolic pathways

#### 3.3.1 CD39 & CD73

The homeostasis of ADO metabolism depends on the precise coordination of ADO-generating enzymes and ADO-clearing enzymes, which jointly regulate ADO levels. In the TME, the aberrant expression and activity alterations of these key enzymes weakens ATP-mediated immune stimulation and enhances ADO-mediated immune suppression, ultimately synergistically promoting the formation of immunosuppressive microenvironment. CD39 and CD73, as cell membrane-bound ADO-generating enzymes, are key rate limiting steps in regulating extracellular ADO generation (90). These two phosphatases are abnormally overexpressed in various tumor tissues and convert ATP into ADO through a cascade reaction, resulting in a significant imbalance of ATP/ADO ratio (91–93).

Extensive experimental evidence confirms that targeting CD39/CD73 pathway is central to tumor immune escape. Targeted therapeutic strategies against this pathway, including gene intervention, antibody therapy, and small-molecule inhibitors, have demonstrated significant efficacy across multiple tumor models. Targeted inhibition of CD39 with anti-CD39 antibody under preclinical development can relieve T cell proliferation inhibition and enhance cytotoxic T lymphocyte (CTL)/NK cell toxicity (94). By adopting the CD73 gene knockout strategy in ovarian tumor model, it was found in mouse with normal immune function that CD73 knockout can induce complete tumor regression in all tumor-bearing mice. In contrast, in tumors without knocking down CD73, simple immunotherapy did not show significant efficacy (95). Anti-CD73 monoclonal antibodies, in models such as breast cancer (4T1.2/E0771) (96) and head and neck squamous cell carcinoma (97), not only suppressed primary tumor growth and metastasis by inducing adaptive antitumor immunity but also reversed T-cell exhaustion. In pancreatic ductal adenocarcinoma model, CD73 was identified as a key gene overexpressed in the top 10% and exhibited identical molecular features to the most aggressive and poorest-prognosis squamous/basal subtypes. Delivery of CD73 small-molecule inhibitors through multiple routes significantly inhibited tumor growth (98). The immunoevasive subtype of cervical cancer and the patient subpopulation of CD8^+^ T cells with high CD39 expression, due to enhanced CD39 enzymatic activity leading to excessive ADO production, drive immune evasion and poor prognosis. Targeted inhibition of CD39 not only enhances the cytotoxicity of CD8^+^ tumor-infiltrating lymphocytes (TILs) but also promotes B cell infiltration, thereby amplifying the anti-tumor immune effect of PD-1 blockade (99). Overall, the above studies targeting CD39/CD73 enzyme can effectively reverse immune suppression and improves antitumor immunity, as well as provide multidimensional experimental support for developing subtype-specific treatment strategies.

#### 3.3.2 ADK

As mentioned above, the homeostasis of ADO metabolism depends not only on the regulation of its generation but also requires the coordination of clearing pathways. Among these, ADK is the key ADO-clearing enzyme that catalyze the conversion of ADO to AMP (21). By phosphorylating ADO into AMP, ADK can eliminate excess extracellular ADO and maintain immune balance (100). On the contrary, inhibiting ADK activity or suppressing its expression can lead to an increase in ADO. Therefore, the expression and catalytic activity of ADK is crucial in regulating the ADO phosphorylation reaction. Gu’s group, through gene knockout to decrease ADK expression levels or ablate its function, demonstrated that ADK regulated ADO metabolism maintains the protein arginine methyltransferase 5-catalyzed symmetric dimethylation at the R606 site of the receptor-interacting serine/threonine kinase (RIPK) death domain, thereby inhibiting overactivation of RIPK1 and cell death, and thus preserving hepatic homeostasis (101).

**Figure 3:**
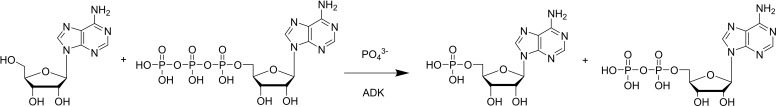
Schematic diagram of phosphate-promoted ADK catalyzed phosphoryl transfer reaction.

For ADK activity, the structural characteristics of its active site directly determine substrate binding efficiency and reaction kinetics (102–104). However, in the TME, high concentrations of ADO inhibit enzyme activity by occupying the active sites of ADK, hindering the binding of ATP to the enzyme, reducing catalytic efficiency, and ultimately suppressing enzymatic activity (105, 106). Research has demonstrated that under physiological/neutral or weakly acidic conditions, phosphate ions can form stable ternary complexes with ADO and ADK through hydrogen bonding, thereby restoring ADK’s catalytic activity and promoting the conversion of ADO to AMP (107, 108). Based on this mechanism, exogenous phosphate supplementation to activate ADK and promote ADO phosphorylation provide potential strategy for regulating ADO levels (**Figure 3**).

Building on this theoretical foundation, material-mediated studies has been conducted to regulate ADK activity. Enzymatic kinetic analysis confirmed that the release phosphate from black phosphorus (BP) (109, 110) and calcium phosphate (CaP) nanomaterials (71) can restore ADK catalytic activity and accelerate the conversion of ADO to AMP in the simulated TME. This research provides a novel intervention strategy that integrates material innovation with enzyme-specific regulation of the ADO metabolic pathway, ultimately achieving ADK enzyme activity regulation and reducing the accumulation of ADO.

#### 3.3.3 ADA

In addition to ADK, ADA also constitutes a key line of defense for clearing ADO. ADA primarily catalyzes the conversion of ADO to INO in the purine metabolic pathway (111, 112). When ADA expression is downregulated or its activity is inhibited, it can also lead to ADO accumulation (113). Its activity abnormalities are closely associated with the occurrence and development of various immune-related diseases (114–116). S. Bagheri and colleagues systematically analyzed dynamic changes in ADA activity under various pathological conditions, elucidating the specific molecular mechanisms by which ADA inhibition contributes to disease pathogenesis (117). Targeting ADA maybe expected to a promising strategy for modulating ADO metabolism and diseases treatment.

There are few reports on intervention strategies for ADA regulation. Currently, known intervention agents include Daidzin, erythro-9-(2- hydroxy-3-nonyl) adenine (EHNA), insulin and polyethylene glycol coupled adenosine deaminase 2 (PEGADA2). Daidzin was reported to exhibit high specificity and strong binding affinity for ADA2, inducing conformational changes in the dimerization domain of ADA2 while leaving the structure of ADA1 unchanged, suggesting that it may serve as a specific therapeutic agent for modulating ADA in the TME (118). After EHNA treatment in cervical cancer cells (119) or insulin treatment (120) in various tissues, ADA activity shows significant decrease trend. PEGADA2 effectively suppress tumor growth and influence immune responses within the tumor microenvironment by targeting ADA activity (121). Furthermore, the strategy of regulating ADA expression via genetic engineering to achieve intratumoral ADO clearance has now begun to be reported in relevant studies. For example, researcher has encoded ADA into an oncolytic herpes simplex virus targeted to human HER2 and constructed engineered ADA carrying an ectopic signal peptide, aiming to enhance its enzymatic secretion efficiency and achieve effective clearance of intratumoral adenosine (122). A genetically engineered strain of Escherichia coli Nissle 1917 that expresses ADA on its surface under hypoxic conditions was reported to achieve *in situ* ADO elimination in tumors via genetic engineering (123).

### 3.4 Targeting ADOR signaling pathway

The physiological effects of ADO exhibit diversity, depending on the implicated receptor subtype, its location, and the tissue circumstances (38). Therefore, targeting ADOR to enhance antitumor immune response or reshape the TME has become a highly promising cancer treatment strategy. Researchers have used ADO as the core to simulate or block its function, and combine the subtype differences of ADOR to screen for corresponding agonists and antagonists. Based on multidimensional strategies such as *in vitro* functional evaluation, computer-aided design, *in vivo* pharmacological validation, and structural biology analysis, a series of highly active and selective candidate molecules have been successfully developed and extensively evaluated in preclinical models and clinical trials (4, 37, 124).

For A2AR, the agonist HENECA (125, 126) influences the immune response within the tumor microenvironment by increasing AC level and intracellular cAMP and suppressing p38 MAPK and activating transcription factor-2 (ATF-2) phosphorylation. While A2AR antagonist, such as TP455 (127), ZM241385, CPI-444 (128), PBF-509 (129, 130), AZD4635 (131), restore immune response and enhance the effectiveness of immunotherapy by the decreasing AC level and cAMP production transduction mechanisms. For A2BR, the antagonists including PSB1115 (132), PSB603 (133), ATL801 (45), PBF-1129 (134) block A2BR to modulate metabolic TME and immunosuppression, ultimately suppressing tumor growth and metastasis. In addition, AB928 (135, 136) and Etrumadenant (137), as dual A2A/A2B antagonists, also play an important role in cancer immunotherapy and ultimately exert anti-tumor effects by blocking immunosuppressive and pro-tumor signals.

All in all, current research has thoroughly investigated the pharmacological properties of different ADOR subtypes, their agonist/antagonist effects, efficacy and safety profiles in preclinical models, as well as the progress and challenges of existing drugs in clinical trials (1, 28, 33, 58, 138, 139), aiming to systematically identify key bottlenecks in the development of ADOR-targeted therapeutics, including issues such as insufficient receptor selectivity, tumor heterogeneity, dynamic changes in the microenvironment, and the synergistic or antagonistic interactions with other immunotherapies.

## 4 Combined modulations of ADO-mediated immunosuppression

Although small-molecule inhibitors targeting CD39 and CD73, or directly blocking ADOR have demonstrated certain therapeutic potential in recent years to alleviate the ADO-mediated immunosuppression. However, these strategies still face significant challenges, including short drug circulation half-life, rapid metabolic clearance, inability of single-target to comprehensively regulate ADO-mediated immunosuppression. To address the issues of the multi-step complexity, target heterogeneity, and limitations of single interventions, combined strategies for ADO-mediated immunosuppression is conducted through synergistic intervention in different metabolic nodes, ultimately enhancing anti-tumor immunity or reversing pathological states (140, 141). Based on this principle, various combinations have been developed, such as “enzyme/ADOR regulation and microenvironment regulation”, “enzyme/ADOR regulation and immune checkpoint”, and “enzyme/ADOR regulation and chemotherapy/radiotherapy”.

For the combined strategy of microenvironment regulation and enzyme regulation, CaP@Fe-MOFs designed and constructed to simultaneously tackle the dual challenges of ADO metabolism and the hypoxic TME. This material promotes the phosphorylation of ADO by ADK, accelerating the conversion of ADO to AMP. Meanwhile, oxygen was generated via Fenton-like reaction to ameliorate the hypoxic TME, thereby reducing the production of ADO form its source and alleviating immunosuppression (71). For the combined strategy of enzyme/ADOR regulation and immune checkpoint, ADO accumulation in the TME is reduced by inhibiting ADO-producing enzymes or activating ADO-degrading enzymes. Simultaneously, combining immune checkpoint inhibitors can alleviate T cell exhaustion and double weaken the immune suppression (142, 143). For example, CD73 inhibitor AB680 combined with anti-PD-1 therapy effectively elicits anti-tumor immune response, ultimately limiting tumor progression and potentiating therapeutic efficacy (144). Patients in recurrent epithelial ovarian cancer (EOC) with combination immunotherapy of oleclumab/anti-CD73 and durvalumab/anti-PD-L1 revealed that CD14^+^CD16^-^ myeloid cells increased (145). In an orthotopic metastatic ovarian cancer mouse model, the combination of anti-CD73 with anti-OX40 significantly increased cytotoxic T-cell infiltration, decreased tumor-promoting immune cells, and simultaneously enhance antibody-mediated immune responses (146). Targeting ADOR in combination with PD-1/PD-L1 antibodies was also reported to overcome immunosuppression (37). Specifically, CD38 suppresses T-cell function via ADOR signaling. The combination of anti-CD38 therapy or ADOR antagonists with anti-PD-L1 effectively overcomes PD-1/PD-L1 resistance (147, 148). For the combined strategy of enzyme/ADOR regulation and chemotherapy/radiotherapy, by using ADOR antagonists to block the inhibitory signals of ADO on immune cells, while combining with radiotherapy, chemotherapy, and oncolytic viruses (86), this strategy utilizes immunogenic cell death to release tumor antigens and activate adaptive immunity. Clinical trials have demonstrated ADOR ligands can significantly enhance anticancer efficacy, particularly when combined with chemotherapy (149). For ease of reference, we have systematically sorted out key information of combined modulations of ADO-mediated immunosuppression in recent years such as combined strategy, experimental model, and related experimental results (**Table 1**). Altogether, based on the advantages and limitations of various anti-tumor treatment methods, multimodal collaborative strategies not only alleviate immunosuppression and enhance immune responses, but also provide effective strategy for precision tumor treatment.

**Table 1:** Research on the combined regulations for ADO-mediated immunosuppression, along with their functional molecule, drug doses, routes of administration and primary outcomes.

Combined strategy	Functional molecule	Drug doses	Routes of administration	Experimental model	Primary outcomes	References
ADK & TME	Phosphate & Fe-MOFs	10 mg kg^−1^	Intratumoral injection	4T1 tumors	Alleviating the Ado-mediated immunosuppressive response and achieving tumor suppression.	(71)
CD73 & TME & PD-1	CD73 inhibitor (AB680) & ZIF-8	10 mg kg^−1^	Intravenous injection	Non-small cell lung cancer	Improving the therapeutic efficacy of PD-1 blockade, leading to robust tumor growth inhibition and prolonging survival of mice.	(150)
CD73 & A2AR	anti-CD73 mAb (clones TY/23 and 2C5) & A2AR inhibitor (SCH58261)	250 μg & 1 mg kg^−1^	Intravenous injection	Melanoma; AT-3 mammary carcinoma; SM1WT1 BRAF mutated melanoma	Limiting tumor initiation, growth, and metastasis.	(151)
	anti-CD73 mAb (2c5mIgG1, murine IgG1) & A2AR inhibitor (AZD4635)	10 mg kg^−1^ &50 mg kg^−1^	Intraperitoneal injection	Pancreatic ductal adenocarcinoma	Inhibiting tumor growth and reducing metastatic burden. Blocking the ADO pathway improved the efficacy of combinations of cytotoxic agents or immunotherapy.	(152)
A2AR & PD-L1/CTLA-4	A2AR antagonist (AZD4635) &PD-L1 mAb (durvalumab)	50 mg kg^−1^ & 10 mg kg^−1^	Intraperitoneal injection	Melanoma; Colonic cancer; Mouse Fibrosarcoma	Reducing tumor burden and enhances anti-tumor immunity. Treatment of AZD4635 with PD-L1 led to decreased tumor volume correlating with enhanced CD103^+^ function and T cell response.	(153)
	siRNA	/	/	CT26 colon cancer	Enhancing antitumor responses via the downregulation of PKA, SHP2, and PP2Aα signaling pathways.	(154)
CD73 & PD-1/PD-L1	CD73 inhibitor (AB680) & PD-1-blocking antibody (29F.1A12)	200 μg & 20 mg kg^−1^	Intraperitoneal injection	C57BL/6J mice	Increasing the vaccine-induced expansion of self-specific CD4^+^ T cells	(155)
	CD73 inhibitor (AB680) & PD-1-blocking antibody (αPD-1)	200 μg mL^-1^ & 200 μg mL^-1^	Tail vein injection	4T1 tumor	Potentiating effective tumoricidal immunity and activates long-lasting immune memory effects	(89)
	CD73 inhibitor (AB680) & PD-1 blocking antibody	10 mg kg^−1^ & 6 mg kg^−1^	Intraperitoneal injection	Colitis-associated cancer model	Improving the anticancer function of immunosuppressed cells such as Treg and exhausted T cells, and reducing Malat1high Treg and M2 macrophages.	(156)
	CD73 mAb (Oleclumab) & PD-L1 mAb (durvalumab)	5–40 mg kg^−1^ & 10 mg kg^−1^	Intravenous injection	stage III non-small-cell lung cancer; advanced colorectal cancer; pancreatic ductal adenocarcinoma	PACIFIC trial demonstrated an acceptable safety profile, with encouraging improvements in PFS and ORR. COAST showed higher ORR and prolonged PFS, longer overall survival.	(157–159)
	CD73 inhibitor (SHR170008) & anti-PD-1 mAb	10 mg kg^−1^ & 5 mg kg^−1^	Intravenous injection	EMT6 mouse breast tumor	Synergistically enhancing antitumor immunity and biomarkers in response.	(160)
A2AR & chemotherapy/photothermal	A2AR inhibitor (SCH58261) & photothermal Immunotherapy	1 mg kg^−1^	Intravenous injection	4T1 tumor	Significantly inhibiting the primary tumor, inhibits distal tumor growth, and prevents lung metastasis.	(87)
	A2AR inhibitor (CPI-444) & temozolomide	5 mg kg^−1^ & 5 mg kg^−1^	Intravenous injection	Orthotopic glioma	Reversing the immunosuppressive feedback signaling pathway of the adenosinergic axis.	(88)
CD73 & chemotherapy/radiotherapy	CD73 knockdown (sh RNA) & Doxorubicin	0.0625 mg & 0.1 mg	Intravenous injection	Glioma	Significantly inhibited postoperative glioma recurrence and progression.	(161)
	siRNA CD73 & Temozolomide	10 μg kg^−1^ & 5 mg mL^−1^	Intraperitoneal injection	Glioblastoma	Reducing tumor size, decreased ADO levels and increasing the sensitivity to temozolomide.	(162)
	Anti-CD73 mAb (clone TY/23) & Oxaliplatin	100 μg/mouse & 5 mg kg^−1^	Intraperitoneal injection	Colon adenocarcinoma	CD73 blockade enhanced oxaliplatin-induced ATP release, promoting DC maturation and immune cell infiltration. The risk of colorectal cancer lung metastasis was decreased.	(163)
	CD73 siRNA & Paclitaxel	/	/	MDA-MB-231 breast cancer	CD73 suppression enhanced paclitaxel’s cytotoxic effects, promoting apoptosis and inhibiting cell migration.	(164)
	CD73 inhibitor (AmPCP) & AmGd-NPs	4 mg kg^−1^ & [Gd] = 1.5 mg kg^−1^	Intravenous injection	4T1 breast cancer	Preventing the conversion of extracellular ATP to ADO and promoting DC maturation.	(165)
	Anti-CD73 mAb (clone 2C5) & X-ray	10 mg kg^−^ & 12Gy	Intraperitoneal injection	Colon MC38 tumor	Improving tumor response to radiotherapy.	(166)
	Anti-CD73 mAb (clone TY/23) & radiotherapy	10 mg kg^−^ & 8 Gy	Subcutaneous injection	Colorectal cancer	Enhancing the antigen-presenting ability of DCs and activating tumor antigen-specific CD8^+^ T cells.	(167)
	CD73 knockdown (sh RNA)/anti-CD73 & radiotherapy	100 μg/mL) & 20 Gy	Intraperitoneal injection	4T1 tumor	Promoting DC infiltration of irradiated tumors.	(168)

## 5 Conclusion and prospects

During tumor development, metabolic and microenvironmental abnormality drives ADO accumulation, the ADO level in the TME significantly increases compared to normal physiological levels. By activating ADOR signaling pathways, ADO suppresses tumor antigen presentation, T cell activation and infiltration, and cytotoxic T lymphocyte-mediated tumor cell killing, thereby exerting immunosuppressive effects and promoting tumor immune evasion. Targeted interventions of ADO metabolism-mediated immunosuppression have emerged as crucial breakthrough direction. Due to the disruption of the dynamic balance between ADO generation and clearance, coupled with the persistent maintenance of an immunosuppressive state in the TME, single-target strategies often exhibit limited efficacy. The combination therapy strategy become an effective approach to overcome the inherent defects of single-target and improve treatment efficacy in cancer immunotherapy, such as “enzyme/ADOR regulation and microenvironment regulation”, “enzyme/ADOR regulation and immune checkpoint”, and “enzyme/ADOR regulation and chemotherapy/radiotherapy”. In addition to gene intervention, antibody therapy, and small molecule inhibitors, responsive nanomaterials are also rapidly developing in these combined strategies. A series of TME-responsive nano-delivery systems have been constructed to achieve targeted drug (inhibitor, antagonist, agonist, etc.) enrichment and controlled release, as well as *in situ* oxygen generation strategies to improve immunosuppressive TME. Combination strategies that integrate enzyme/ADOR/microenvironment regulation with other metabolic intervention (169) are equally indispensable.

In combination therapy targeting ADO metabolism-mediated immunosuppression, tumor subtype specificity will also be an important factor to consider. ADO metabolic characteristics of different tumor subtypes exhibit significant heterogeneity (170, 171). As mentioned in section of CD39 & CD73, across different tumor subtypes, significant heterogeneity exists in the expression levels and catalytic activities of key metabolic enzymes. This difference directly affects the efficacy and risk of side effects of drugs targeting the pathway of ADO metabolism (172, 173). In addition, ADOR are all G protein-coupled receptors and their signaling pathways exhibit crosstalk. Depending on the receptor subtype, tumor subtype, and the TME, these pathways play diverse roles in tumor progression, sometimes promoting or inhibiting tumor growth (174–176). For tumor patients with overexpression of related genes and phenotypic features, in-depth analysis of ADOR subtype and tumor subtype is a prerequisite for constructing subtyping-guided targeting strategies in clinical trials. To sum up, in view of the advantages and limitations of various anti-tumor treatment methods, optimizing combined therapeutic strategies in conjunction with subtype specificity is the key path to achieve precise regulation.

**Figure 4:**
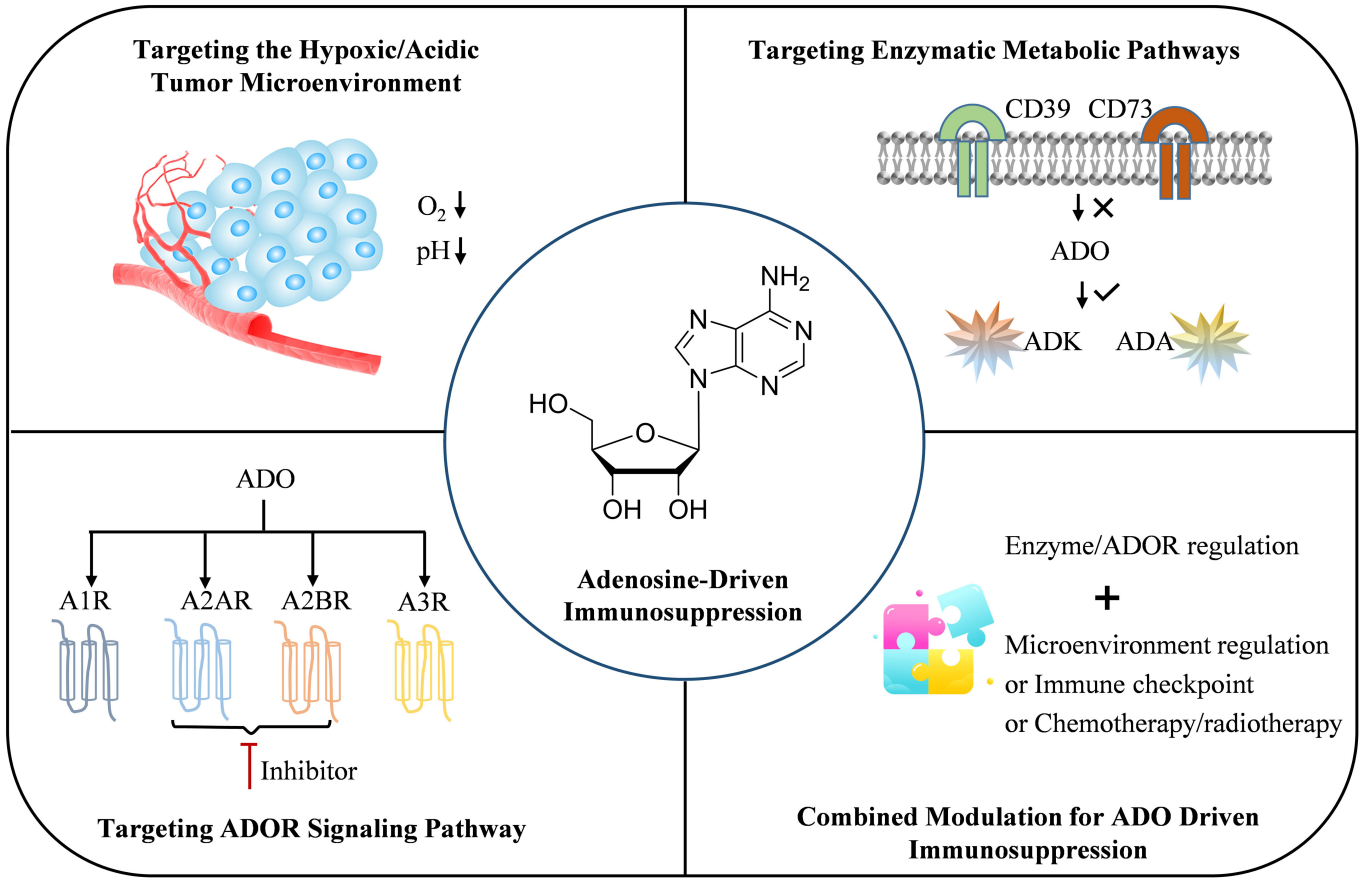
Develop a comprehensive strategy for immune suppression driven by ADO metabolic abnormalities through “multi-enzyme combinatorial targeting, microenvironment-responsive delivery, precision subtyping and multi-pathway synergy”.

This review systematically summarizes the key aspects of targeting ADO-mediated immunosuppression, including the feature of TME, enzymes involved in ADO metabolism (e.g., CD39/CD73/ADK/ADA), and ADOR interventions. Additionally, the necessity of comprehensively regulating ADO metabolism and the immune microenvironment through multi-level coordinated interventions is also explored, as well as the latest combined regulatory strategies. All in all, given the immunosuppressive state of the microenvironment, the complexity of the ADO pathway, and its impact on multiple cell types, it profoundly affects immune cell function and tumor progression. Further integration of “microenvironment responsive delivery, multi-enzyme combination targeting, precise subtypes, and multi-pathway synergy” (**Figure 4**) is needed to deepen our understanding of tumor immune evasion phenomena and provide feasible pathways for immunometabolic therapy.

## Glossary

AC: Adenylate cyclase
ADA: Adenosine deaminase
ADK: Adenosine Kinase
ADP: Adenosine diphosphate
ADPR: ADP ribose
ADO: Adenosine
ADOR: Adenosine receptors
AK: Adenylate kinase
AMP: Adenosine monophosphate
ATP: Adenosine triphosphate
Bc@AZTF: Bacterial cells@ZIF-90&AB680& Tannic acid&iron-polyphenol
BP: Black phosphorus
CaP: Calcium phosphate
cAMP: Adenosine 3',5'-cyclic monophosphate
CD4: Cluster of differentiation 4
CD8: Cluster of differentiation 8
CD16: Cluster of differentiation 16
CD38: Cluster of differentiation 38
CD39: Ectonucleoside triphosphate diphosphohydrolase
CD73: Ecto-5'-nucleotidase
CNT/ENT: Concentrative nucleoside transporter/Equilibrative nucleoside transporter
cNT: Cytoplasmic nucleotidase
COX2: Cyclooxygenase-2
CREB: cAMP response element-binding protein
CTLA-4: Cytotoxic T-lymphocyte-associated protein 4
CTL: Cytotoxic T lymphocyte
Cx: Connexin hemichannels
DCs: Dendritic cells
EHNA: Erythro-9-(2- hydroxy-3-nonyl) adenine
EPAC: Exchange protein activated by cAMP
Fe-MOFs: Iron-based metal-organic frameworks
FOXP3: Forkhead box P3
HER2: Human epidermal growth factor receptor 2
HIF-1α: Hypoxia-inducible factor 1α
IL-6: Interleukin-6
IL-8: Interleukin-8
IL-10: Interleukin-10
IL-12: Interleukin-12
IDO: Indoleamine 2,3-dioxygenase
INO: Inosine
LAG3: Lymphocyte activating 3
MDSCs: Myeloid-derived suppressor cells
NAD: Nicotinamide adenine dinucleotide
NADP: Nucleotide diphosphokinase
NK: Natural killer
ORR: Objective response rate
PD-1: Programmed cell death protein 1
PD-L1: Programmed death-ligand 1
PEGADA2: polyethylene glycol coupled adenosine deaminase 2
PFS: Progression-free survival
PKA: Protein kinase A
SAH: S-adenosylhomocysteine
SAHH: SAH hydrolase
STAT3: Signal transducer and activator of transcription 3
TCM: T central memory cells
TGF-β: Transforming growth factor-β
TILs: Tumor-infiltrating lymphocytes
TME: Tumor microenvironment
TNF-α: Tumor necrosis factor-α
Treg: Regulatory T cell
